# Interview with Sabato D’Auria, Section Editor for the Chemical Biology section in *BMC Biochemistry*

**DOI:** 10.1186/1471-2091-14-14

**Published:** 2013-06-14

**Authors:** Sabato D’Auria

**Affiliations:** 1 Institute of Protein Biochemistry, Via Pietro Castellino, 111, 80131, Naples, Italy

## 

Sabato D’Auria is currently a senior scientist with a tenured position within the National Research Council of Italy as the head of the Laboratory for Molecular Sensing at the Institute of Protein Biochemistry CNR, Naples, Italy. Dr D’Auria is also invited Professor at the INRS-Institute Armand-Frappier, Laval, Quebec, Canada.

Dr D’Auria is a well known biochemist whose scientific interests are focused on the identification, isolation and structural characterization of proteins, and their application as a specific probe for the design of advanced optical biosensors. Dr. D’Auria’s laboratory uses some of the most advanced biophysical techniques available in their research, including Surface Plasmon Resonance, Fluorescence Correlation Spectroscopy for single molecule detection, time-resolved fluorescence, and near infrared fluorescence spectroscopy.

In this article we find out a little more about the field of chemical biology and discuss what Dr D’Auria would like to see submitted to his section.

## How did you first become interested in biochemistry?

After my thesis was published, which focused on the “synthesis of oligonucleotides”, I was asked to consider a post-doc position at the Institute of Protein Biochemistry, CNR, Naples to study proteins and enzymes isolated from thermophilic micro-organisms. I found these biomolecules fascinating and powerful since I felt that they could represent an “ideal” protein model to understand the relationship between protein folding, stability, flexibility, and function. Later on, as I came to understand more about this class of proteins, I became interested in an important biotechnological application of them; using them as specific and stable probes for the design of advanced optical biosensors.

## Why is it an exciting time to be involved in chemical biology in particular?

What is exciting is that more than other research areas, in chemical biology the fields of chemistry and biology are strongly intertwined and so researchers must have an interdisciplinary approach. Chemical biology involves the application of chemical techniques, tools, or compounds to the study and manipulation of biological systems. The recent huge progresses in bioinformatics and probe chemistry, such as the possibility to study protein dynamics in a timescale of microseconds, and the commercial availability of new fluorescence chemical probes with an emission in the near infra-red region, open promising avenues to study the role of proteins in cell function. In fact, today it is possible to highlight new molecular interactions inside the cell (also at the single molecule level) and consequently to infer the existence of new regulatory mechanisms of proteins in cell life.

## What challenges and developments can we expect to see in the next few years in this field?

In my opinion, one of the major challenges in chemical biology is the labeling of endogenous proteins in their natural environment by synthetic probes. In the next few years, we hope that new near-infrared sensing proteins for total body imaging techniques will be discovered, as well as the synthesis of new near infrared probes with high quantum yield. The near-infrared band represents a spectral window where the human body is transparent. It is well known that hemoglobin in the blood absorbs visible wavelengths shorter than 650 nm, while water molecules absorb wavelengths above 900 nm. It appears evident that the possibility to have a protein with an emission window in the region between 650 nm and 900 nm means it could be possible to develop an advanced human body marker, since the near infrared radiations penetrate easily into and out of tissues.

## How can novel chemical biology research contribute to meeting these challenges?

The future of biology in the coming years is affected by the difficulty of studying extremely complex systems. Bioinformatics and systems biology will play an important role in this area, and chemical biologists will contribute to meeting these challenges in two ways. Firstly, they will have to make an effort to understand and interact with researchers in the fields of bioinformatics and systems biology. Secondly, they will have to fully exploit the capacity to develop and characterize new fluorescence probes (both for small molecules and macromolecules) that will be able to help cell biologists monitor different components within organisms and manipulate these *in vivo* and in the whole animal.

## How can chemical biology advances be applied to other fields?

In my opinion the area of research around bio-sensing and in particular the design of novel, highly performing biosensors is one of the most promising applications of the advances in chemical biology. In fact, the discovery of novel proteins to use as bio-markers and/or the synthesis of new chemical probes to use for labeling proteins and antibodies, are essential for moving towards a rapid diagnosis directly on real matrices such as whole blood.

The chemical biology section will consider submissions covering the application of chemistry to the investigation of biological processes and systems including the biosynthesis and metabolism of natural compounds, drug design, and complex molecular rearrangements occurring in living organisms.

## Are there any particular papers you would like to see submitted to your section?

Manuscripts addressing various aspects of the design and development of new biosensors based on innovative concepts would be very welcome as this is an important and growing area of research at the moment. Manuscripts submitted to the chemical biology section will benefit from the open access format that the journal provides, ensuring that this work will be widely and quickly disseminated after publication, with no cost to readers.

